# Inhibitory Effect and Mechanism of Dancong Tea from Different Harvesting Season on the α-Glucosidase Inhibition In Vivo and In Vitro

**DOI:** 10.3390/foods13244183

**Published:** 2024-12-23

**Authors:** Rourou Wen, Xianghua Chai, Pingping Wang, Kegang Wu, Xuejuan Duan, Jiasi Chen, Tong Zhang, Liya Zeng

**Affiliations:** 1 School of Chemical Engineering and Light Industry, Guangdong University of Technology, Guangzhou 510006, China; 18316011109@163.com (R.W.); xhchai813@sohu.com (X.C.); wukegang2003@163.com (K.W.); wandxjbb@163.com (X.D.); supercat1109@163.com (J.C.); fsezzz308364445@163.com (T.Z.); 18316124100@163.com (L.Z.); 2 Guangdong Province Laborary of Chemistry and Fine Chemical Engineering Jieyang Center, Jieyang 515200, China

**Keywords:** Phoenix single dancong tea, catechin monomers, EGCG, enzyme inhibition

## Abstract

Tea polyphenols have been reported to decrease the rate of starch hydrolysis by inhibiting α-glucosidase. However, the effect of the tea harvesting season and the structure of catechin monomers on the inhibitory activity of α-glucosidase is not understood. In this study, the inhibitory effect and underlying mechanism of four seasons of Dancong tea against α-glucosidase were investigated by in vivo and in vitro experiments, multi-spectroscope and molecular dynamic. The Dancong tea harvested in spring and winter showed a stronger inhibitory effect on α-glucosidase due to a higher content of catechin, especially EGCG ((-)-epigallocatechin-3-gallate). The results of in vivo and in vitro experiments showed that EGCG and ECG ((-)-epicatechin-3-gallate) with a higher content of gallate and hydroxyl groups exhibited a stronger inhibitory effect on starch hydrolysis, rise of postprandial blood glucose and activities of α-glucosidase compared to EGC ((-)-epigallocatechin) and EC ((-)-epicatechin). These gallate and hydroxy groups were more effective in interacting with the amino acid residues in the active site of α-glucosidase, leading to structural changes in the enzyme. Certainly, the inhibitory effect of Dancong tea on α-glucosidase explains one of the mechanisms by which it helps alleviate diabetes; the other hypoglycaemic mechanisms of Dancong tea will be further explored.

## 1. Introduction

Fenghuang Dancong tea is a very old tea which dates back to a period 700 years ago [1] and is cultivated on Fenghuang Mountain in the east of Guangdong Province, China. Dancong tea has become increasingly popular worldwide due to its unique sensory characteristics (such as aroma, taste, and color), health benefits, and cultural significances [2]. Tea is universally recognized to have a natural abundance of phenolic constituents, endowed with various health-promoting attributes, including antioxidant, anti-inflammatory, and antitumor activities, making it a valuable contributor to overall wellbeing. In addition, tea polyphenols have been found to reduce carbohydrate digestibility and slow postprandial blood glucose elevation by inhibiting the activity of α-glucosidase. Based on our comprehensive understanding, the environmental factors, notably climate, humidity levels, soil moisture content, and ambient temperature, play pivotal roles in influencing the growth patterns and development of tea plants. Hence, the teas harvested in different seasons have different enzymatic and chemical properties, resulting in different tea polyphenols composition and content. Diabetes, a common chronic disease, has affected the standard of living of 536.6 million people globally in 2021 [3]. Previous studies have reported that tea polyphenols could reduce carbohydrate digestibility and slow postprandial blood glucose elevation by inhibiting the activity of α-glucosidase to prevent and treat diabetes [4]. Nevertheless, research about the differences of the inhibition ability to α-glucosidase between the tea harvested from four different seasons is very rare, which has a detrimental impact on the production and advancement of Dancong tea.

Currently, the inhibitory effects of tea polyphenols on α-glucosidase and their mechanism of action are the focus of research and have great practical significance. As the main component of tea polyphenols, catechins have been widely reported to be safe, efficient, and side-effect-free α-glucosidase mechanism agents [5]. Modern pharmacological research has shown that tea polyphenols can interact with starch hydrolases to regulate blood sugar through hydrogen and hydrophobic bonds [6]. The phenolic hydroxyl moieties possess the ability to interact with hydroxyl, amino, and carboxyl functionalities present in starch hydrolases, consequentially modifying their structural configurations and biological functionalities. This modification process diminishes the enzymes’ efficacy in digesting starch, thereby achieving a notable reduction in starch digestibility. Wu [7] reported that the ECG inhibition mechanism for α-glucosidase is related to the reaction with four key amino acid residues of α-glucosidase: Lys156, Ser157, Arg315, and Asp352, thereby reducing the enzyme’s activity. However, there are few studies in the literature on the types and contents of catechins obtained from different harvesting seasons of Dancong tea, and the effect of structure of the catechin monomers on the inhibitory activity α-glucosidase and anti-diabetes function.

In this work, the inhibitory effect and underlying mechanism of four seasons of Dancong tea against α-glucosidase were investigated by in vitro and in vivo digestion experiments. The inhibitory effects, inhibitory type, and conformational changes of the four major catechin monomers (EGCG, ECG, EGC, and EC) with different number and substitution sites of hydroxyl and galloyl groups on α-glucosidase were systematically investigated by kinetic analysis, fluorescence burst spectroscopy, synchronous fluorescence spectroscopy, and circular dichroism. Furthermore, the intermolecular interaction mechanisms and the inhibition mechanism on the catechin monomers of different structure with catechin monomers were revealed using molecular docking and molecular dynamic techniques. This research illuminated the distinctive effect of the timing of harvest on the chemical makeup and in vitro anti-glycemic digestion potential of Dancong tea, elucidating a seasonal influence on its bioactive properties. Furthermore, the interaction mechanism and structure-activity relationship of the catechin monomers on the inhibitory ability of α-glucosidase were identified.

## 2. Materials and Methods

### 2.1. Materials

The fresh leaves of Fenghuang Dancong tea were harvested from the Xingning Town, Meizhou city, Guangdong Province, China in spring, summer, autumn and winter of 2022. The samples were prepared after the process of tea-greening, sun-greening, shaking (2 min each time, once every 2 h, a total of 7 times), greening, twisting, initial baking (30 min at 70 °C), baking (2 h at 120 °C), and reheating (8 h at 90 °C).

HPLC-grade acetonitrile and methanol were purchased from Aladdin Biological Co., Ltd. (Shanghai, China). p-nitrophenyl-α-D-galactopyranoside (*p*NPG) was provided by Shanghai Yuanye Bio-Technology Co., Ltd. (Shanghai, China). α-glucosidase (26.3 U/mg) and absorbance were purchased from Shanghai Yuanye Bio-Technology Co., Ltd. (Shanghai, China). EGCG, ECG, EGC, and EC (purity, ≥98%) were obtained from Beijing Psaitong Biotechnology Co., Ltd. (Beijing, China). Glucose and corn starch were provided by Shanghai Macklin Biochemical Co., Ltd. (Shanghai, China).

### 2.2. Determination of Catechin Monomers

A total of 1.0 g of freshly harvested tea leaves, collected across the four seasons, underwent extraction using 50 mL of water at a temperature of 90 °C for a duration of 10 min. Following this, the extract was subjected to filtration through a nylon membrane with a pore size of 0.22 μm for purification. A K2025 HPLC system (Shandong Wooking Instrument Co., Ltd., Jinan, China) comprising a Kromasil 100-5-C18 chromatogram column (250 × 4.6 mm, 5 μm) and a C18 guard column (250 × 4.6 mm, 5 μm; Phenomenex, Gemini, Torrance, CA, USA) was prepared. The HPLC separation process employed solvent A, comprising acetonitrile, and solvent B, a 0.02% aqueous solution of formic acid, as the dual-component mobile phase for effective separation. A volume of precisely 10 μL of tea extract was introduced into the HPLC apparatus, operated under a stable flow rate of 1.2 milliliters per minute. The system employed gradient elution for optimal separation, and the UV detector was set to a wavelength of 278 ns. The quantitative analysis of the catechin monomers was conducted using the appropriate calibration curves, which exhibited a high degree of linearity (R^2^ > 0.990).

### 2.3. In Vitro Digestion Properties

Starch digestibility was measured following the previous method with minor modifications [8]. Corn starch (1.0 g) was dispersed in 50 mL of sodium acetate buffer (0.1 mol/L, pH 5.2), and then mixed with 1.0 mg/mL or 2.0 mg/mL of EGCG/ECG/EGC/EC solution, stirring for 30 min (2000 rpm, 90 °C), and cooled to 37 °C. Three mL mixtures of porcine pancreatic a-amylase (15 U/mL) and a-glucosidase (10 U/mL) were added, and incubated at 37 °C for 120 min. The concentration of hydrolyzed glucose was quantitatively assessed via the DNS colorimetric approach at various time intervals, encompassing 0, 10, 20, 30, 40, 80, 100, and 120 min. The DNS (100 μL) was added to the sample then the mixture was boiled for 5 min, and the absorbance was detected at 540 nm. The starch hydrolysis was calculated by the following formulas:

(1) $$\mathrm{SH}\;(\%)=\frac{0.9\times G}{TS}\times 100\%$$

where the ratio of 0.9 signifies the comparative molecular weights between starch and glucose. The quantity of glucose generated through enzymatic breakdown is denoted as G (g). Prior to enzymatic digestion, the total starch content in the sample is referred to as TS (g).

### 2.4. Animal Experiments and Glucose Tolerance Test

C57BL/6J male mice (n = 16, 7-week-old) were acquired from the Hunan SJA Laboratory Animal Co., Ltd. (Changsha, China), and maintained in a specific pathogen-free laboratory at the Animal Center of Guangdong Pharmaceutical University. The animals were maintained in a meticulously controlled environment, featuring a 12-h light–dark cycle maintained at a temperature of 23 ± 1 °C and a humidity level of 55% ± 5%. They had continuous access to an abundant supply of food and sterile, potable water, ensuring optimal conditions for their well-being and experimental integrity. All experiments were approved by the Experimental Animal Ethical Committee (approval no. gdpulacspf 2017505).

After fasting overnight, 16 mice were randomly assigned into four groups and fasted overnight before oral gavage of the mixture of EGCG/ECG/EGC/ECC (50 mg/kg·bw) and soluble starch solution (1 g/kg·bw). The blood glucose levels were quantified via tail clipping at 0, 30, 60, 90, and 120 min following the administration of glucose in all animals. The area under the curve (AUC) were calculated. Mice were euthanized by carbon dioxide inhalation at the end of the experiment. The CO_2_ was administered via ‘gradual fill’. It was infused into the euthanasia chamber at a rate of 10% to 30% per minute.

### 2.5. α-Glucosidase Inhibitory Activity Assay

The inhibitory potential against α-glucosidase was evaluated following previously established protocols, albeit with subtle refinements [9]. Firstly, α-glucosidase (2.0 U/mg) was dissolved in 0.1 mg/mL PBS (pH = 6.8) solution and pre-incubated at 37 °C for 10 min. Then 50 μL α-glucosidase solution, 50 μL different concentrations of EGCG/ECG/EGC/EC (0.08, 0.16, 0.32, 0.64, 0.80, 1.00 mg/mL) and 50 μL *p*NPG (5 mM) were mixed thoroughly and incubated at 37 °C for 20 min. Finally, 100 μL of Na_2_CO_3_ (0.2 M) was used to terminate reaction, and the activity of α-glucosidase was evaluated according to the amount of p-nitrophenol released from the substrate *p*NPG at 405 nm. Acarbose was used as the positive control. The inhibition rate was expressed as

(2) $$\mathrm{Inhibition rate}\;(\%)=\frac{A_{0}-A_{1}}{A_{0}}\times 100\%$$

In this context, A_0_ signifies the absorbance value obtained from the control experiment, where 50 μL of phosphate-buffered saline (PBS) was utilized in lieu of samples. The ultimate outcome is expressed in terms of the half-maximal inhibitory concentration (IC_50_), measured in milligrams per milliliter. Conversely, A_1_ represents the absorbance recorded for the test samples.

### 2.6. Analysis of α-Glucosidase Inhibition Kinetics

A kinetic analysis of the impact of EGCG/ECG/EGC/EC on α-glucosidase was undertaken, leveraging the Lineweaver–Burk equation as the method of choice for elucidating the rate-determining factors and their interrelationships [10]. The different concentrations of *p*NPG (0.25, 0.50, 1.00, and 1.25 mM) with different inhibitor concentrations (0.00, 0.08, 0.32, and 0.80 mg/mL) were incubated with α-glucosidase at 37 °C for 5 min. Utilizing an enzyme marker, the absorbance was recorded at a wavelength of 405 ns, with measurements taken at a frequency of once per minute.

(3) $$\frac{1}{V}=\frac{K_{m}}{V_{max}}\left(1+\frac{\left[I\right]}{K_{i}}\right)\frac{1}{\left[S\right]}+\frac{1}{V_{max}}\left(1+\frac{\left[I\right]}{K_{is}}\right)$$

where [I] and [S] represent the concentrations of the enzyme and substrate, respectively. The Michaelis–Menten constant (*K_m_*) and the maximum reaction velocity (*V_max_*) pertain specifically to the α-glucosidase enzyme. Additionally, *K_i_* and *K_is_* denote the dissociation constants, with *K_i_* referring to the binding of the catechins with α-glucosidase, and *K_is_* to the interaction between α-glucosidase and the *p*NPG.

### 2.7. Fluorescence Quenching Experiments

The α-glucosidase solution (0.5 U/mL) of 50 μL was titrated by continuous addition with 50 μL different concentrations of EGCG/ECG/EGC/EC (0.00, 0.08, 0.16, 0.32, 0.64, 0.80, and 1.00 mg/mL) at 304 K or 310 K. After equilibration for 5 min, the fluorescence spectra at 300–400 nm were measured using an RF6000 spectrofluorometer (Shimadzu, Kawasaki, Japan) at an excitation wavelength of 280 nm; the excitation and emission bandwidths were 10 nm. To decipher the underlying mechanism of fluorescence quenching, the Stern–Volmer equation was employed as a tool for comprehensive analysis [11]. The fluorescence data employed in this study underwent correction via an established formula, aiming to mitigate and eliminate potential inner filter effects that could confound the analysis [12].

(4) $$F=F_{0}e^{(A_{1}+A_{2})/2}$$

In this context, *F*_0_ signifies the raw experimental fluorescence data, whereas *F* denotes the fluorescence data post-correction. Correspondingly, *A*_1_ and *A*_2_ are the ultraviolet absorbance values of the inhibitor, measured specifically at the excitation and emission wavelengths, respectively.

In addition, the fluorescence quenching parameters of EGCG/ECG/EGC/EC with α-glucosidase were calculated based on Equations (5)–(7):

(5) $$\frac{F_{0}}{F}=1+[Q]K_{SV}=1+[Q]K_{q}\tau_{0}$$

(6) $$lg\frac{F_{0}-F}{F}=lgK_{a}+nlg[Q]$$

(7) $$lgK_{a}=-\frac{∆H}{2.303RT}+\frac{∆S}{2.303R}$$

where *F*_0_ and *F* denote the intensity of fluorescence without or with quencher. *K_SV_* and *K_q_* are the fluorescence quenching constant and quenching rate constant, respectively. K_a_ is the binding constant, and *τ*_0_ (10^−8^ s) is the fluorescence lifetime of α-glucosidase. N indicates the amount of fluorescence quenching binding sites, and [Q] stands for quencher concentration. ΔH and ΔS represent the enthalpy and entropy changes of the reaction system, respectively.

### 2.8. Circular Dichroism (CD) Analysis

The CD spectra of α-glucosidase (0.4 U/mL) and EGCG/ECG/EGC/EC (0.00, 0.04, and 0.80 mg/mL) were acquired from 190 to 260 nm on a Chirascan spectrometer (MOS 450, Bio-logic Corp., France) at 298 K. The entire analysis was conducted under a nitrogen-purged atmosphere to ensure consistent conditions. A quartz cuvette with a path length of 0.05 mm was utilized for precise optical measurements. Then the data were calculated by CDPro-SELCON3 software to analyze the changes of protein secondary structure (α-helices, β-sheet, β-turn, and random coiling).

### 2.9. Synchronous Fluorescence Spectra Experiments

Synchronous fluorescence spectra of α-glucosidase (0.4 U/mL) and EGCG/ECG/EGC/EC (0.00, 0.02, 0.04, 0.08, 0.16, and 0.64 mg/mL) were acquired with intervals (Δλ) of 15 and 60 nm between excitation and emission wavelength. After reaction with 5 min, the fluorescence spectra were measured using an RF6000 spectrofluorometer (Shimadzu, Japan) under the conditions of 250–350 nm and 335 K.

### 2.10. Molecular Docking

Molecular docking was carried out employing AutoDock 4.2 software to investigate the interaction between α-glucosidase and the catechins. The crystal structure of α-glucosidase was obtained from the Protein Data Bank (http://www.rcsb.org; PDB ID: 3AJ7), accessed on (11 October, 2023), and the 3D structures of EGCG, ECG, EGC, and EC were obtained from the PubChem database (https://pubchem.ncbi.nlm.nih.gov/), accessed on (11 October 2023), and then optimized using Gaussian 16 software (Version 1.1). Before the docking process, the molecules of water and ligands of α-glucosidase were removed, and the PDBQT coordinate for both the modeled α-glucosidase and EGCG/ECG/EGC/EC were generated. The center box was defined by the dimensions of 126 × 126 × 126 Å, with the grid spacing of 0.375 Å. Furthermore, the molecular docking simulations were executed utilizing the Lamarckian genetic algorithm (LGA) as the computational framework. Following an extensive iteration of 100 runs, the docking configuration exhibiting the most favorable, i.e., lowest binding energy, was chosen for delving into the mode of action by which EGCG, ECG, EGC, and EC interact with and modulate α-glucosidase activity. The binding mode between EGCG/ECG/EGC/EC and α-glucosidase were visualized using PyMOL 1.6.

### 2.11. Molecular Dynamic (MD) Simulations

Molecular dynamic simulation was performed by GROMACS 4.5.6. to investigate the interaction between α-glucosidase and EGCG/ECG/EGC/EC. Amber 14sb and Amber GAFF were selected as the protein and the small-molecule force field, respectively. To simulate the aqueous environment, the TIP3P water model was incorporated into the α-glucosidase–EGCG/ECG/EGC/EC system. Then the counterions (Na^+^ and Cl^−^ ions) were added to neutralize the simulation systems. The neutralized configurations underwent energy minimization utilizing the steepest descent algorithm. Subsequently, the minimized systems were equilibrated under isothermal–isovolumetric (NVT) and isothermal–isobaric (NPT) conditions, maintaining a constant temperature of 300 K and a pressure of 1 atm for a duration of 20,000 ps to ensure stability. Ultimately, unrestrained production simulations spanning 100 ns were executed. To assess the stability of the complexes, root mean square deviation (RMSD) graphs were generated based on these 100-nanosecond production runs [13].

### 2.12. Statistical Analysis

Results were presented as a mean value ± SD (standard deviation, n = 3), and statistically analyzed using SPSS v. 27 software. Analysis of variance (ANOVA) was carried out by Dunnett’s test, where *p* < 0.05 was assumed to be statistically significant.

## 3. Results and Discussion

### 3.1. Effect of Tea Harvesting Season on α-Glucosidase Inhibition

**Figure 1:**
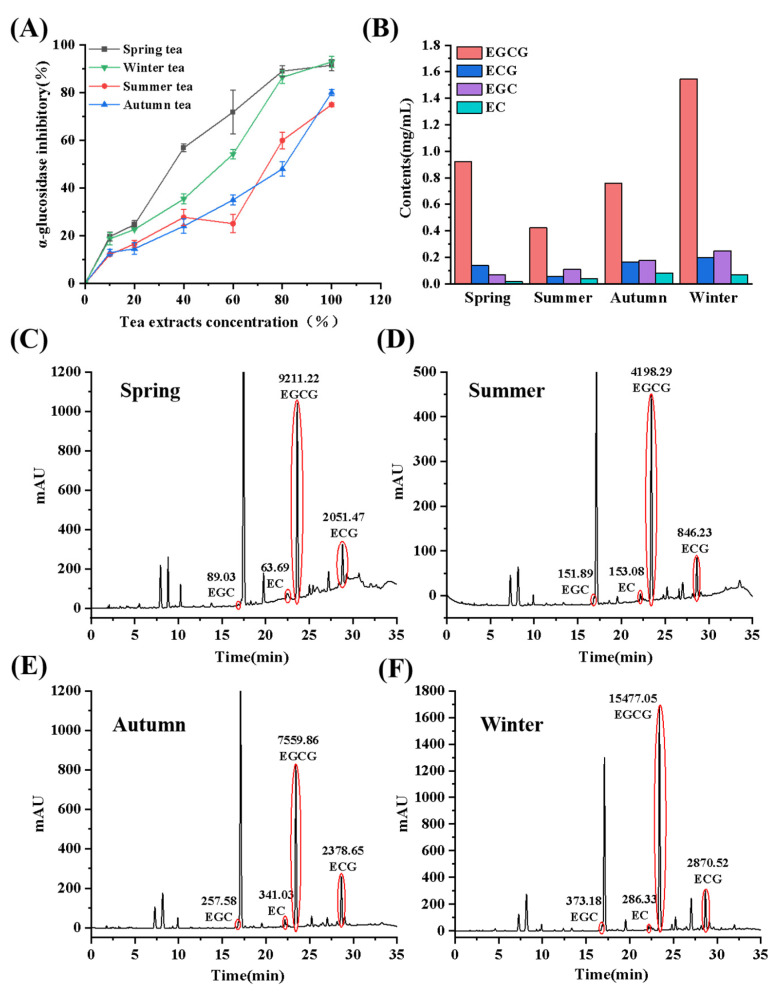
Effect of tea harvesting season on α-glucosidase inhibition (**A**); Contents of the catechin monomers in Dancong four season tea (**B**); HPLC analysis chromatogram of the catechin monomers in spring tea (**C**), summer tea (**D**), autumn tea (**E**), and winter tea (**F**).

As shown in Figure 1A, the inhibition rates for four seasons of tea extracts against α-glucosidase were ranked as follows: spring tea ≈ winter tea > autumn tea ≈ summer tea. The Dancong tea harvested in spring and winter showed the higher inhibitory effects on α-glucosidase. Based on previous studies, the difference in the inhibitory effect of seasonal tea on α-glucosidase was due to the varying types and contents of the catechin monomers. The contents of the four main catechin monomers (EGCG, ECG, EGC, EC) in spring tea, summer tea, autumn tea, and winter tea (Figure 1C–F) were determined by HPLC, and the monomer contents of the catechins were calculated according to the peak areas. The tea harvested in winter exhibited the highest total contents (2.063 mg/mL) of the four catechin monomers, and EGCG was the most abundant catechin monomer in all four seasons of tea (Figure 1B). In addition, winter tea (1.5461 mg/mL) contained the highest amount of EGCG, followed by spring tea (0.9224 mg/mL), autumn tea (0.7580 mg/mL), and summer tea (0.4234 mg/mL); and summer tea (0.0574 mg/mL) contained the smallest amounts of ECG, compared to spring (0.1407 mg/mL), autumn (0.1633 mg/mL), and winter (0.1973 mg/mL) teas. The fewer contents of the esterified catechins in summer tea and autumn tea were related with their lower inhibitory ability on α-glucosidase. On the contrary, the winter tea and spring tea could effectively inhibit the activity of α-glucosidase due to their relatively high content of esterified catechins, especially the higher content of EGCG. To further investigate the accuracy of this hypothesis, the inhibitory effects and mechanism of the four catechin monomers (EGCG, ECG, EGC, and EC) on α-glucosidase were studied by in vivo and in vitro digestion, multispectral techniques, and molecular dynamics simulations.

### 3.2. Effect of Catechin Monomers on the α-Glucosidase Inhibitory Activity In Vivo and In Vitro

**Figure 2:**
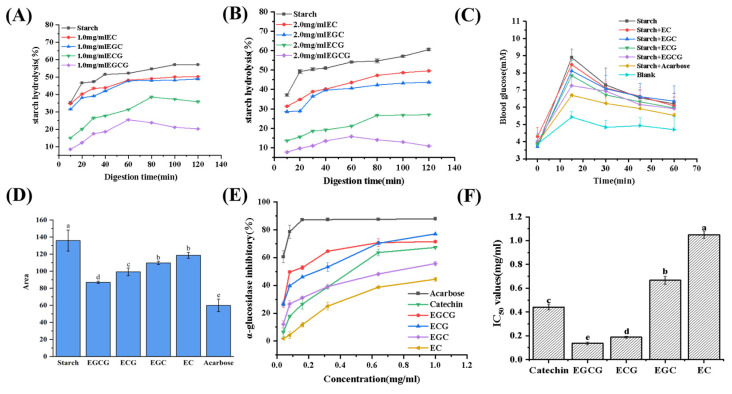
Effect of the catechin monomers on in vitro digestion of starch (**A**,**B**); in vivo postprandial blood glucose content (**C**) and α-glucosidase inhibition (**E**); the calculated area under the curve of glucose content (**D**) and IC50 values (**F**). Different letters (a–e) in Figure 2D,F indicate significant differences (*p* < 0.05).

As shown in Figure 2, the addition of the catechin monomers significantly reduced the hydrolysis rate of starch, decreased postprandial blood glucose content, and increased the α-glucosidase inhibition ability, which indicated that the four catechin monomers possessed the ability of inhibiting starch hydrolysis, glucose absorption, and α-glucosidase activity. The addition of the catechin monomers of 2.0 mg/mL caused hydrolysis rates of corn starch to remain essentially stable at 120 min, with values of 10.85% (EGCG), 27.01% (ECG), 43.74% (EGC), and 49.55% (EC) (Figure 2B). EGCG and ECG exhibited a stronger inhibitory effect on starch hydrolysis compared to EGC and EC. In addition, the postprandial blood glucose curve in vivo (Figure 2C) and the area under the curve (Figure 2D) showed that EGCG had the best effect in slowing down the increase in postprandial blood glucose levels, followed by ECG, EGC, and EC. After gavage of starch, the postprandial blood glucose level of mice indirectly reflected the activities of α-glucosidase, which was necessary for the starch hydrolysis to increase blood glucose levels [14]. Furthermore, the inhibition rates and kinetics (Table 1) of EGCG, ECG, EGC, and EC on α-glucosidase were determined and showed that the four monomers had a dose-dependent inhibitory effect against α-glucosidase (Figure 2E). EGCG and ECG consistently showed the higher inhibitory effects on α-glucosidase than EGC and EC in the concentration range of 0.08–1.00 mg/mL, and the IC_50_ values of the four catechin monomers against α-glucosidase were ranked as follows: EGCG (0.14 ± 0.01 mg/mL) < ECG (0.19 ± 0.01 mg/mL) < EGC (0.67 ± 0.04 mg/mL) < EC (1.05 ± 0.03 mg/mL) (*p* < 0.05). The lowest IC_50_ value attained by EGCG demonstrated its superior inhibitory potency towards α-glucosidase, concurring with both in vivo findings regarding postprandial glycemic control and in vitro evaluations of starch digestion inhibition.

The results of inhibition kinetics of the catechin monomers against α-glucosidase showed that all the straight lines intersecting at the origin (Figure S1), which suggested that the inhibition process of the four catechin monomers was reversible, and the modes of combination between α-glucosidase and the four catechin monomers were non-covalent interactions [15]. Lineweaver–Burk plots were employed as an analytical tool to delve into the kinetic characteristics and the nature of the interaction between the catechin monomers and α-glucosidase, thereby elucidating the underlying mechanisms (Figure S2). The slope of the line decreases as the catechin concentration increases, and all lines are compared to the second quadrant, which suggests that the four catechin monomers has a mixed-type inhibition of α-glucosidase [16]. In addition, kinetic inhibition parameters for α-glucosidase were calculated based on Lineweaver–Burk plots; the results are shown in Table 1. The Km value of α-glucosidase was 0.0680 mM, and the Vmax (the maximum reaction rate) value was 0.0464 ∆A405/min. The *V*_max_ decreased continuously with the concentration of the catechin monomers increasing, and the order of decreasing *V*_max_ value was EGCG > ECG > EGC > EC. In addition, the *K*_i_ values were in descending order: ECG < EGCG < EGC < EC, indicating that EGCG and ECG were more likely to combine and inhibit the activity of α-glucosidase. Meanwhile, the *K*_i_ values of EGCG (1.2765 mg/mL) and ECG (1.1052 mg/mL) were lower than their *K*_is_ of EGCG (1.7166 mg/mL) and ECG (1.6051 mg/mL), respectively, which indicated that α-glucosidase bound more easily with EGCG and ECG than *p*NPG (p-Nitrophenyl-α-D-galactopyranoside) [17]. By contrast, *K*_i_ > *K*_is_ for EGC and EC indicated that α-glucosidase preferred to bind with substrates, not EGC and EC. The results of the inhibition kinetics suggested that EGCG and ECG reacted more readily with α-glucosidase than EGC and EC, leading to a more pronounced decrease in enzyme activity. As previously reported, the extra gallate groups and -OH group in the structures of EGCG and ECG might enhance their ability to interact with α-glucosidase through non-covalent bonding, leading to an increased inhibitory effect [18].

### 3.3. Effect of Catechin Monomers Type on the Structure of α-Glucosidase

#### 3.3.1. Fluorescence Quenching of α-Glucosidase by Catechin Monomers

**Figure 3:**
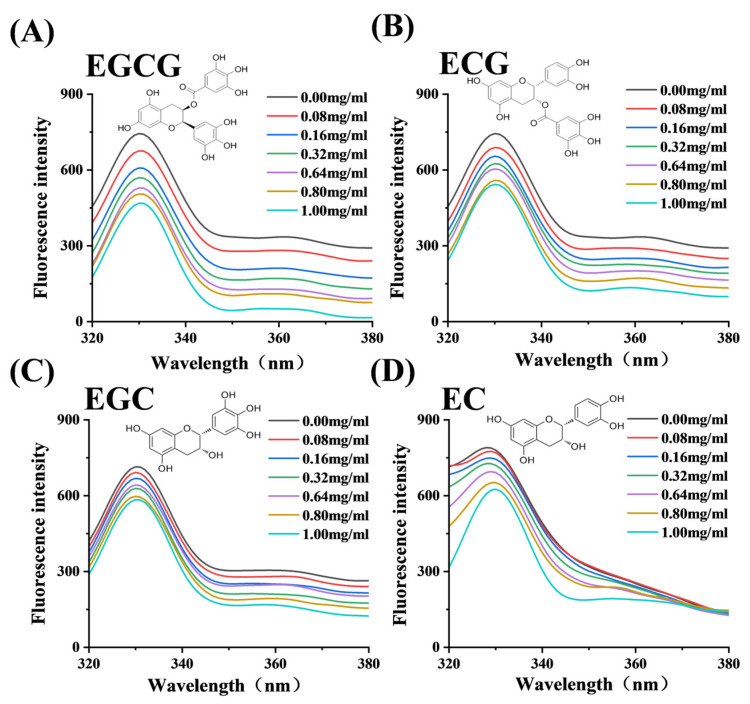
Fluorescence quenching effects on α-glucosidase by EGCG (**A**), ECG (**B**), EGC (**C**), EC (**D**).

The affinity and thermodynamic parameters of α-glucosidase binding to the catechin monomers were investigated by fluorescence quenching. As shown in Figure 3, the fluorescence intensity of α-glucosidase decreased gradually with the concentration of the four catechin monomers increasing, indicating that the four monomers could quench inherent fluorescence of α-glucosidase. The Stern–Volmer equation at different temperatures showed good linearity (Figure S3), and the fluorescence quenching parameters were listed in Table 2. The values of *K*_SV_ decreased significantly with the increasing temperature, suggesting that the mechanism by which catechin monomers quench the fluorescence of α-glucosidase was a static process [19]. In addition, the observed negative ΔH values and positive ΔS values indicate that the primary interactions between the catechin monomers and α-glucosidase are attributable to hydrogen bonding, alongside significant contributions from hydrophobic forces [17]. Specifically, ECG and EGC had larger *K*_a_ values than EGCG and EC, suggesting the stronger binding ability with α-glucosidase residues. However, the binding stability together with the binding location affected the inhibitory ability on α-glucosidase, and the binding of monomers to amino acid residues close to the active center of the enzyme was more beneficial in reducing the activity of the enzyme [20], which would be further explored by molecular docking and dynamics.

#### 3.3.2. Secondary Structural Changes of α-Glucosidase

**Figure 4:**
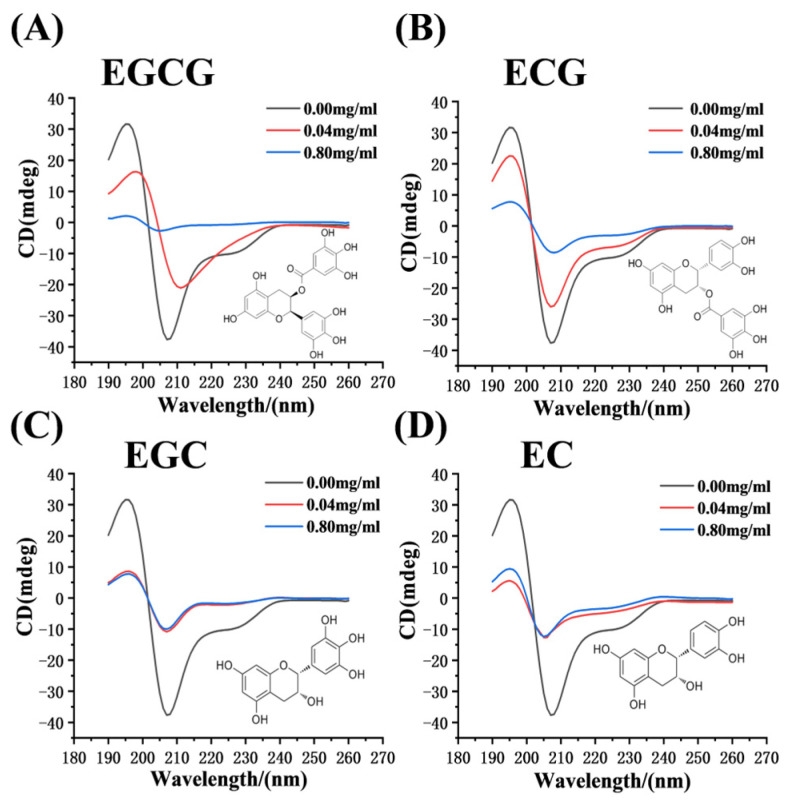
The CD spectra of α-glucosidase with various concentrations of EGCG (**A**), ECG (**B**), EGC (**C**), EC (**D**).

As shown in Figure 4, two major negative bimodal bands at 209 and 228 nm were observed in the CD spectrum of α-glucosidase, which were typical of an α-helix structure arising from peptide bond π → π* and n → π* leaps. The distinct positive absorption peak at 195 nm represented the characteristic peak of the β-sheet. The secondary structure contents of α-glucosidase were then calculated using a software program (Table 3). With the addition of a catechin monomer up to a concentration of 0.80 mg/mL, there was a tendency for both the α-helix and β-sheet of α-glucosidase to decrease, with the β-turn being more stable and irregular curling varying accordingly. Specifically, EGCG showed the most obvious decline in the content of the α-helix and β-sheet, with a reduction rate of 47.20 ± 0.264% and 34.63 ± 0.586%, respectively. By contrast, EGC and EC (the reduction in the α-helix content of 17.30 ± 0.815% and 15.20 ± 0.692%, respectively) had less effect on the secondary structure content of α-glucosidase. The lower amount of the α-helix and β-sheet after the addition of the catechin monomers in the α-glucosidase might result in a more relaxed network structure overall, which led to better access of the catechin monomers to the enzyme’s active center, further preventing the binding of enzyme to the substrate resulting in reduced α-glucosidase activity [21]. The results of the experiments showed that EGCG and ECG altered the secondary structure content of α-glucosidase more than EGC and EC, further leading to greater inhibition.

#### 3.3.3. Structural Changes of Amino Acid Residues of α-Glucosidase

**Figure 5:**
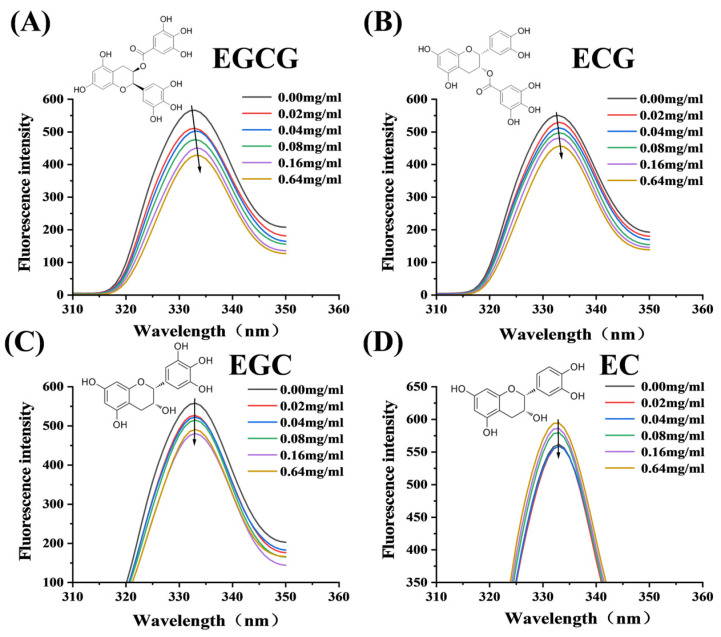
The effect of EGCG (**A**), ECG (**B**), EGC (**C**), EC (**D**) on the synchronous fluorescence spectra of α-glucosidase at Δλ = 15 nm.

The microenvironmental alterations of tyrosine (Tyr) and tryptophan (Trp) residues in α-glucosidase could be discerned through an analysis of the synchronous fluorescence spectra at Δλ = 15 and 60 nm, respectively [22]. As the concentration of the catechin monomers increased, the synchrotron fluorescence intensities of Tyr and Trp in α-glucosidase decreased. The maximum fluorescence emission wavelength of EGCG and ECG for Tyr showed red shift, but that of EGC and EC had no significant shift for Tyr (Figure 5). Whereas the maximum fluorescence emission wavelength monomers of the four catechin monomers showed no obvious shift for Trp (Figure S4). The results revealed that the polarity of the microenvironment surrounding Tyr residues was enhanced and the hydrophobicity was reduced by EGCG and ECG. In addition, the order of fluorescence intensity quenching of Tyr residues was EGCG > ECG > EGC > EC, indicating that EGCG and ECG might contribute more conformational structure changes on α-glucosidase than EGC and EC. These results were in agreement with the functional results that EGCG and ECG exhibited a stronger inhibitory effect on the activity of α-glucosidase, and the winter tea with the highest EGCG and ECG contents showed the strongest ability of α-glucosidase inhibition.

### 3.4. Molecular Docking and Dynamics Analysis

**Figure 6:**
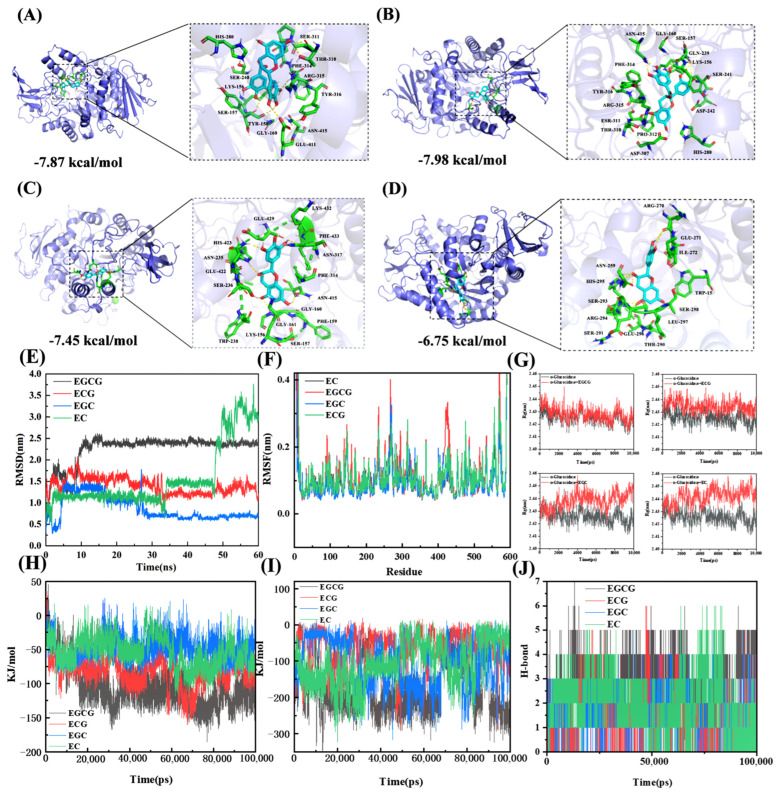
The optimal 3D docking conformation of EGCG (**A**), ECG (**B**), EGC (**C**), EC (**D**) with α-glucosidase. The molecular dynamic results of RMSD (**E**), RMSF (**F**), Rg (**G**), Lennard–Jones potential (**H**), Coulomb interaction energy (**I**), hydrogen bond profile (**J**) of α-glucosidase–EGCG/ECG/EGC/EC complex.

Figure 6A–D showed the molecular docking conformations with the lowest binding energy selected after 100 dockings of EGCG, ECG, EGC, and EC with α-glucosidase, respectively. The reaction mechanism of catechin monomers with α-glucosidase were analyzed at the molecular level, and the data for the molecular docking results were organized in Table 4.

The lowest binding energy of EGCG to α-glucosidase was −7.87 kcal/mol (Figure 6A). EGCG was surrounded by 15 amino acid residues of α-glucosidase, of which residues Lys156, Tyr158, His280, Pro312, Phe314, and Arg315 were the active sites of α-glucosidase. EGCG formed six hydrogen bonds with other amino acid residues of α-glucosidase, including residues Thr310, Arg315, Glu411, Tyr158, and Ser157, with residues Arg315 and Tyr158 being at the active site of α-glucosidase. Meanwhile, EGCG also interacted with nine amino acid residues of α-glucosidase through hydrophobized force. Figure 6B demonstrated the interaction of ECG and α-glucosidase with the lowest binding energy of −7.98 kcal/mol among the four monomers. ECG was surrounded by 16 amino acid residues of α-glucosidase, six of which were active site residues, including Lys156, Tyr158, His280, Pro312, Phe314, and Arg315. ECG formed one hydrogen bond with Lys156 of the active site residues, and seven hydrogen bonds with other residues Asn415, Ser157, Ser311, and Ser241 of α-glucosidase. In addition to hydrogen bonding, ECG binds to nine amino acid residues of α-glucosidase by hydrophobic forces. The results were in agreement with the conclusion of the fluorescence quenching described above that catechin monomers bind to glucosidase primarily by hydrogen bonding and hydrophobic forces. Therefore, EGCG and ECG could effectively inhibit enzyme activity in two ways: binding with the active site of the enzyme through hydrogen bonding and hydrophobic forces; binding with the surrounding cavity to cause a hindrance and prevent substrate entry.

The lowest binding energy of EGC and EC was −7.45 and −6.75 kcal/mol, respectively, which was higher than the binding energy of EGCG and ECG with α-glucosidase. The EGC and EC was surrounded by the 16 and 13 amino acid residues of α-glucosidase and formed six and four hydrogen bonds with other amino acid residues. However, none of those amino acid residues was in the active site of α-glucosidase. Consequently, EGC and EC could not directly bind to amino acids in the active site of glucosidase, but relied on hydrogen bonding or hydrophobic forces to bind to residues other than those in the active site to prevent the substrate from entering the active center of the enzyme. The four monomers were ranked by their binding energies with α-glucosidase as follows: ECG < EGCG < EGC < EC, which could be explained that the extra gallate and hydroxyl group of EGCG and ECG increased the likelihood of interaction between the monomers and α-glucosidase active site residues through hydrophobic interaction, hydrogen bonding, and benzene ring conjugation [21,22].

Figure 6E shows that EGCG and ECG equilibrated around 10 ns, and EGC stabilized at 30 ns, but EC still fluctuated significantly at 50 ns. Overall, The stability order of the four catechin monomers bound to α-glucosidase was EGCG > ECG > EGC > EC, which was line with the above results of in vivo and in vitro inhibitory activity of the four catechin monomers on α-glucosidase. The activity of amino acid residues was significantly increased by EGCG, but less affected by the other three monomers (ECG, EGC, EC) (Figure 6F), which further proved that EGCG significantly altered the molecular structure of α-glucosidase. In addition, the differences of Rg value between the α-glucosidase and EGCG was smallest (Figure 6G), and the energy needed for the reaction of the four monomers with α-glucosidase followed this order: EGCG < ECG < EGC ≈ EC (Figure 6H), indicating that the molecular structure of α-glucosidase and EGCG binding was the most tightly packed, followed by ECG, EGC, and EC. Among the four monomers, only EGCG produced a stronger electrostatic charge when reacting with α-glucosidase (Figure 6I), and EGCG showed the more hydrogen bonds number (7) with α-glucosidase than ECG (6), EC (6) and EGC (5) during the MD simulation. These results of molecular simulations showed that EGCG and ECG with more gallate and hydroxyl groups more readily react with α-glucosidase, and formed more stable and tight hydrogen bonding than EGC and EC.

## 4. Conclusions

In the present study, the Dancong tea harvested in spring and winter showed the higher inhibitory levels on α-glucosidase than summer and autumn harvests, which could be explained by the higher total contents of the four catechin monomers, especially EGCG. The results of in vivo and in vitro experiments demonstrated that the catechin monomers could inhibit starch digestion and postprandial blood glucose levels through the inhibitory activity of α-glucosidase. In addition, the order of inhibition of α-glucosidase by the four catechin monomers was EGCG > ECG > EGC > EC. The results of the fluorescence quenching experiments, and molecular dynamics suggested that the catechin monomers interact with α-glucosidase mainly through hydrophobic forces and hydrogen bonding. EGCG and ECG, esterified catechins, with more gallate and hydroxyl groups, more readily react with amino acid residues at the α-glucosidase active site, and formed more stable and tight hydrogen bonding and hydrophobic interaction than EGC and EC. The binding altered the conformation of α-glucosidase, reducing the number of α-helices and β-sheets and making its structure more relaxed. This increased accessibility of EGCG and ECG to the enzyme’s active center preventing the enzyme from binding to its substrate. The results indicated that spring- and winter-harvest Dancong teas with a higher EGCG content exhibited a stronger inhibitory effect on α-glucosidase. The structure–function relationship of the four catechin monomers on α-glucosidase inhibition effects was then illustrated. It provided the theoretical basis for the selection of Dancong tea harvest seasons with anti-diabetic function using catechins as functional components.

## Figures and Tables

**Table 1:** Kinetic parameters for the inhibition of α-glucosidase by EGCG, ECG, EGC, and EC.

	EGCG	ECG	EGC	EC
*K*_m_ (mM)	0.0680			
*V*_max_ (∆A405/min)	0.0464			
*V*_max_ (∆A405/min) (0.16 mg/mL)	0.0255	0.0287	0.0289	0.0252
*V*_max_ (∆A405/min) (0.32 mg/mL)	0.0116	0.0142	0.0163	0.0163
*V*_max_ (∆A405/min) (0.80 mg/mL)	0.0093	0.0123	0.0132	0.0136
*K*_i_ (mg/mL)	1.2765	1.1052	1.5295	1.5793
*K*_is_ (mg/mL)	1.7166	1.6051	1.0789	1.3258

**Table 2:** The quenching constants, binding constants, and thermodynamic parameters between EGCG, ECG, EGC, EC, and α-glucosidase.

	T (K)	Ksv (10³L/mL)	Ka (10³L/mL)	n	logKa	∆H (KJ/mL)	∆G (KJ/mL)	∆S (J/K/mL)
EGCG	331	1.58	1.36	1.18	0.13	−44.76	−846.32	2.42
	335	1.17	1.12	1.05	0.05		−856.01	
ECG	331	2.71	2.18	1.34	0.34	−34.13	−2145.02	6.38
	335	2.66	1.88	1.20	0.27		−2170.53	
EGC	331	2.37	2.21	1.38	0.34	−7.42	−2182.64	6.28
	335	2.23	1.40	1.32	0.15		−2208.93	
EC	331	2.75	1.25	1.12	0.10	−42.4	−614.18	1.73
	335	2.08	1.04	1.02	0.02		−621.09	

**Table 3:** Secondary structure contents of the catechin monomers–α-glucosidase complex with different concentrations.

	Concentrations (mg/mL)	Helix/%	β-Sheet/%	Beta-Turn/%	Rndm. Coil/%
EGCG	0	39.10 ± 0.173	28.10 ± 0.200	16.63 ± 0.058	16.13 ± 0.321
	0.04	31.47 ± 0.058	22.27 ± 0.153	16.97 ± 0.115	29.23 ± 0.321
	0.8	20.63 ± 0.153	18.37 ± 0.115	17.37 ± 0.153	43.60 ± 0.361
Decrease rates (%)		47.20 ± 0.264	34.63 ± 0.586		
ECG	0	39.10 ± 0.173	28.10 ± 0.200	16.63 ± 0.058	16.13 ± 0.321
	0.04	34.43 ± 0.153	26.33 ± 0.252	16.53 ± 0.208	22.60 ± 0.200
	0.8	28.27 ± 0.153	24.40 ± 0.173	16.03 ± 0.306	31.37 ± 0.321
Decrease rates (%)		27.73 ± 0.115	13.16 ± 0.321		
EGC	0	39.10 ± 0.173	28.10 ± 0.200	16.63 ± 0.058	16.13 ± 0.321
	0.04	33.43 ± 0.208	23.40 ± 0.265	12.33 ± 0.208	30.73 ± 0.208
	0.8	32.33 ± 0.415	22.97 ± 0.404	12.33 ± 0.153	32.40 ± 0.179
Decrease rates (%)		17.30 ± 0.815	18.26 ± 0.357		
EC	0	39.10 ± 0.173	28.10 ± 0.200	16.63 ± 0.058	16.13 ± 0.321
	0.04	33.40 ± 0.173	21.07 ± 0.058	16.57 ± 0.058	28.97 ± 0.153
	0.8	33.17 ± 0.115	22.27 ± 0.153	11.67 ± 0.153	32.80 ± 0.265
Decrease rates (%)		15.20 ± 0.692	20.76 ± 0.929		

**Table 4:** Data on the results of molecular docking of the catechin monomers with α-glucosidase.

Monomer	Binding Energy /(kcal/mol)	Amino Acid Residues Number	Active Site Residues	Total Hydrogen Bond Number	Hydrogen Bond Number (Active Site)
EGCG	−7.87	15	6	6	2
ECG	−7.98	16	6	7	1
EGC	−7.45	16	2	6	0
EC	−6.75	13	0	9	0

## Data Availability

The original contributions presented in the study are included in the article/Supplementary Material, further inquiries can be directed to the corresponding author.

## Supplementary Materials

The following supporting information can be downloaded at: https://www.mdpi.com/article/10.3390/foods13244183/s1, Figure S1: The reversibility of four catechin monomers on α-glucosidase; Figure S2: Lineweaver-Burk plots of the catechin monomers; Figure S3: Stem−Volmer diagrams of the catechin monomers; Figure S4: The effect of four catechin monomers on synchronous fluorescence spectra of α-glucosidase at Δλ = 60 nm.
