# Supplementary material for: Inhibitory Effect and Mechanism of Dancong Tea from Different Harvesting Season on the α-Glucosidase Inhibition In Vivo and In Vitro
Source: Foods. 2024 Dec 23;13(24):4183. doi: 10.3390/foods13244183 (PMC11675673; doi:10.3390/foods13244183)
Supplement: Supplementary file 1 [file foods-13-04183-s001.zip › foods-3379113-supplementary.pdf]

Inhibitory effect and mechanism of DanCong tea with different harvesting season on the α-glucosidase inhibition *in vivo* and *in vitro*

Rourou Wen [a], Xianghua Chai [a], Pingping Wang [a,b,*], Kegang Wu [a], Xuejuan Duan [a], Jiasi Chen [a], Tong Zhang [a], Liya Zeng [a]

[a] School of Chemical Engineering and Light Industry, Guangdong University of Technology, Guangzhou, 510006, China.

[b] Guangdong Province Laborary of Chemistry and Fine Chemical Engineering Jieyang Center, Jieyang, China

Corresponding author

*Pingping Wang: piwa@gdut.edu.cn

Figure S1. The reversibility of four catechin monomers on α-glucosidase.

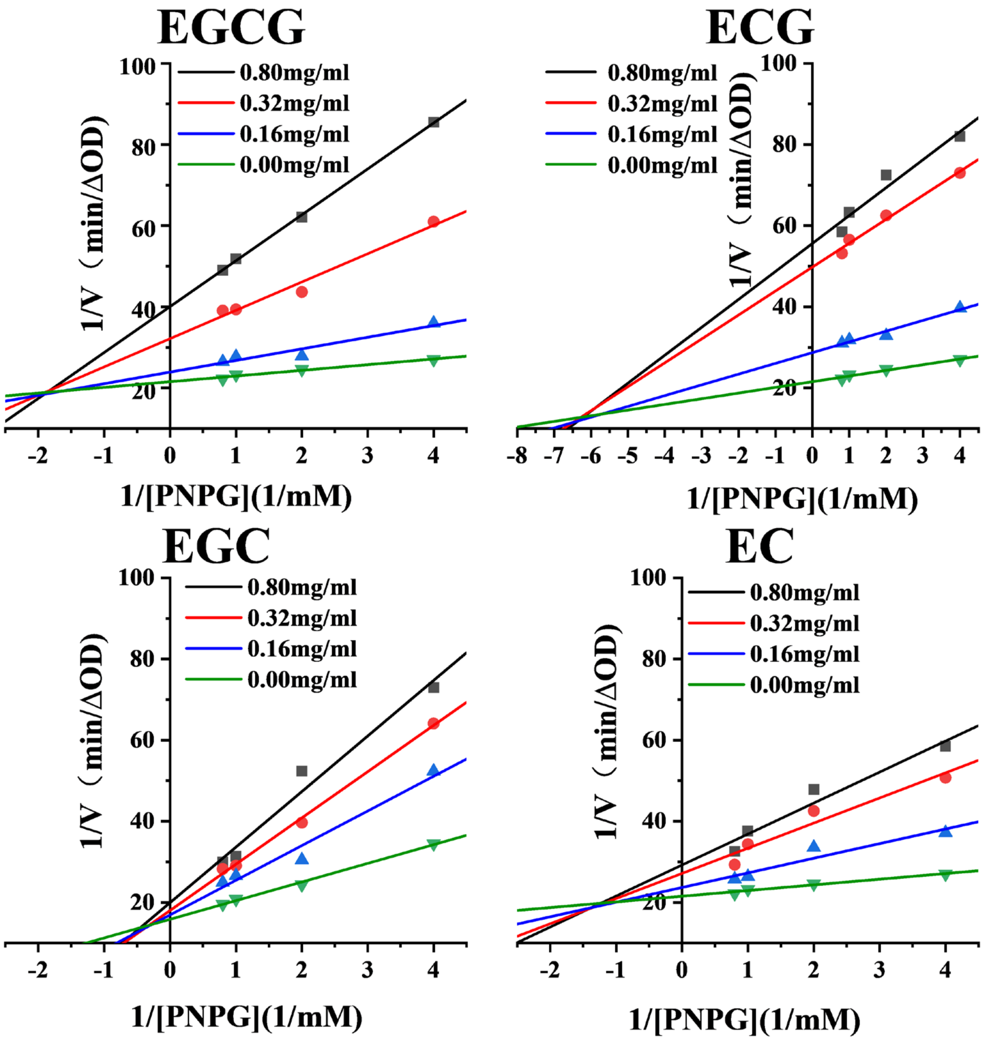

Figure S2. Lineweaver-Burk plots of the catechin monomers.

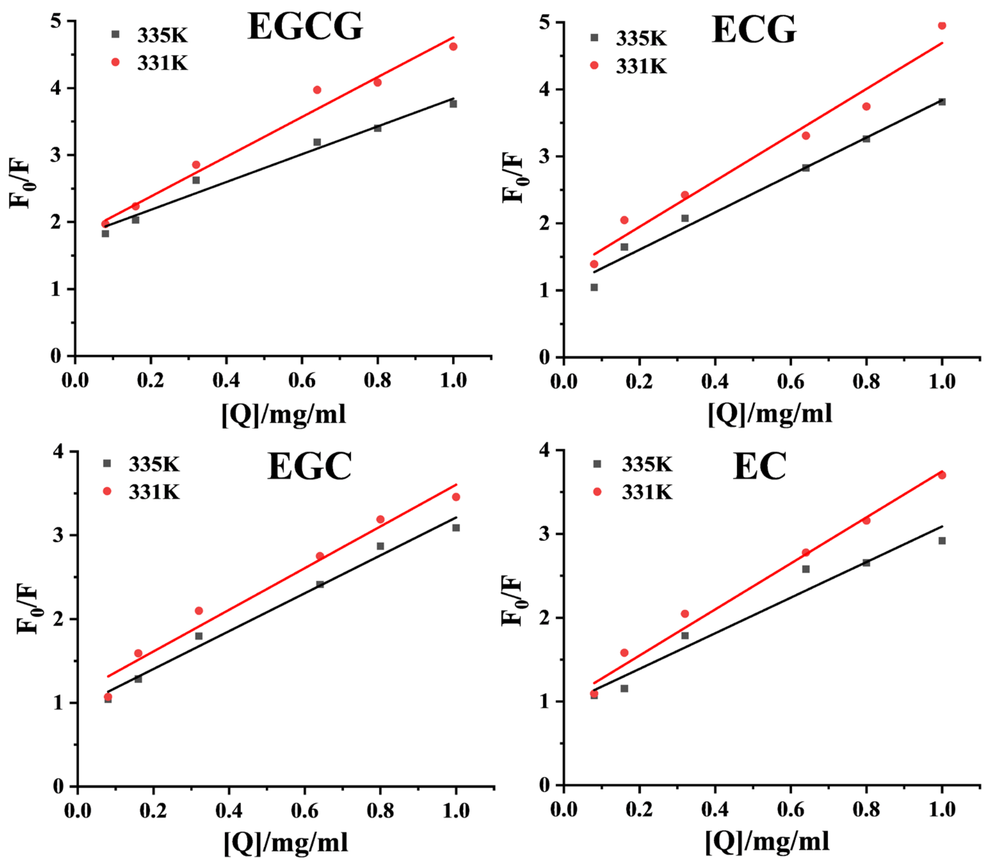

Figure S3.Stem−Volmer diagrams of the catechin monomers.

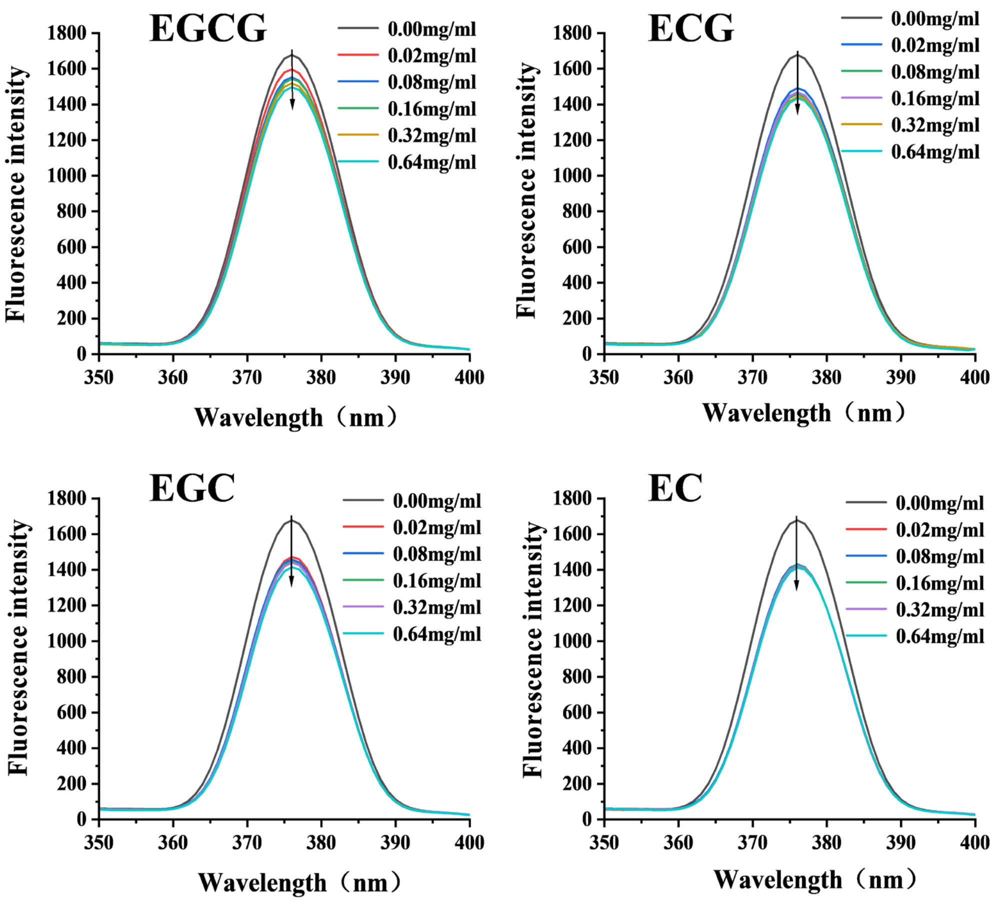

Figure S4.The effect of four catechin monomers on synchronous fluorescence spectra of α-glucosidase at Δλ= 60 nm.
